# “Christian Science” and the Practice of Medicine

**Published:** 1898-12

**Authors:** 


### "Christian Science" and the Practice of Medicine.

A recent issue of the *Sun* contained a most admirable article, headed A Dangerous Cult, apropos of the indictment, in England, of a "Christian Science" woman under whose ministrations Mr. Harold Frederic had died. The writer points out that the most dangerous form of quackery is one founded on pseudo-religious superstition, as is the case with "Christian Science." "If," he says, "a man kills his child in obedience to a fanatical belief that the sacrifice must be made as a duty to God, as Abraham prepared to sacrifice Isaac, and as Jephthah actualy did sacrifice his daughter, according to the best authorities, he is a murderer, and the more dangerous to society because of the very sincerity of his fanaticism." The writer then goes on to show how the devotees of "Christian Science" have multiplied in this country, and how they constitute such an aggressive host as to have practically, according to all appearances, frightened legislative committees in the States of New York and Massachusetts into reporting adversely on bills intended to put a stop to their particular form of quackery, or into taking action equivalent to such a report. He gives extracts from the impious effusions—as profuse as they are meaningless—by which the champions of the sect have effected such intimidation.

What sort of a conscience can a legislator have who suffers his plain common sense to be overdone by the fact that a crowd of fanatics has so overrun his committee room that he feels constrained to hold the sessions in the senate chamber? Yet the "Christian Science" are not by any means the first who have balked wholesome legislation or accomplished what was bad. We have never approved of unnecessary legislation; much less have we advocated it. Perhaps in this instance the bills intended to prevent medical practice by the "Christian Scientists" may prove to be unnecessary. We can only tell by observing how the courts deal with persons who may be prosecuted under existing laws, and it will be interesting to note how the case of the woman indicted in London is disposed of. The "Christian Scientists" contend that they are not practicing medicine within the meaning of the law, but their contention is arrant nonsense. Whenever one of them takes charge of a sick person, to the exclusion of his care at the hands of a duly qualified practitioner, he or she is unquestionably practicing medicine, and should be dealt with summarily, irrespective of the termination of the case.

But we must take things as we find them. Legislative committees, and even legislatures themselves, are overawed by a crowd of blatherskites, unless there is a sufficient force to counteract them, and the counteracting force must be imposing in numbers as well as logically correct. Let us of the medical profession, then, effect such an organization as shall command a respectful hearing. Let not the rank and file leave the rights and welfare of the profession to be looked after by a few of the more prominent of its members, but let the force of numbers be felt. The legally registered practitioners of the State of New York, for example, constitute a force quite capable of stamping out the last vestige of quackery, if only they were properly organized.—Editorial in *New York Medical Journal.*

